# New records of two lycaenid butterfly species (Lepidoptera: Lycaenidae) in China, with the description of a new subspecies

**DOI:** 10.3897/BDJ.9.e69073

**Published:** 2021-06-16

**Authors:** Sixun Ge, Zhuoheng Jiang, Lili Ren, Shaoji Hu

**Affiliations:** 1 College of Forestry, Beijing Forestry University, Beijing, China College of Forestry, Beijing Forestry University Beijing China; 2 School of Science, Westlake University, Hangzhou, China School of Science, Westlake University Hangzhou China; 3 Institute of International Rivers and Eco-security, Yunnan University, Kunming, China Institute of International Rivers and Eco-security, Yunnan University Kunming China

**Keywords:** *Tajuria sekii*, *Drupadia scaeva*, Lycaenidae, Yunnan, new record, new subspecies, Oriental Region

## Abstract

**Background:**

The family Lycaenidae is the second-largest group of butterflies which contains about one third of the known species of Papilionoidea. The genera *Tajuria* Moore, [1881] and *Drupadia* Moore, 1884 are both mainly found in the Oriental and Australian realms. In a very recent expedition to south-west China in Xishuangbanna (Yunnan Province), specimens of *T. sekii* Saito, 2005 and *D. scaeva* (Hewitson, 1869) were collected for the first time, a new subspecies of the former: *T. sekii sisyphus* ssp. nov., is described and illustrated and the latter species comprises the first record of the genus *Drupadia* in China.

**New information:**

A new subspecies of *T. sekii* Saito, 2005, *T. sekii sisyphus* ssp. nov., is described and illustrated. The species *T. sekii* Saito, 2005 and *D. scaeva* (Hewitson, 1869) are first recorded in China and the latter comprises the first record of the genus *Drupadia* in China. Relevant details are presented for the species.

## Introduction

The family Lycaenidae is the second largest family of butterflies, which includes over 6,000 species (Pierce et al. 2002). Lycaenidae harbours a high species diversity and plays an important role in many ecosystems (Sharma and Sharma 2021). The adults are of small size, but brightly coloured and almost always with metallic iridescence on the uppersides, especially in males (Shou et al. 2006).

The genus *Tajuria* contains more than 30 species, most of which have shiny bluish to violet iridescence on the upperside of the wings. This genus is mainly found in the Indo-Malayan Realm, while the Indo-China Peninsula is the centre of species diversity (Corbet 1940).

The genus *Drupadia* contains 12 known species and can be found from northeast Himalaya to the Philippines (Cowan 1974, Shou et al. 2006). To date, there is no official record of this genus in China.

In this contribution, specimens of *T. sekii* Saito, 2005 and *D. scaeva* (Hewitson, 1869) were first collected in Yunnan Province and a new subspecies, *T. sekii sisyphus* ssp. nov., is described and illustrated.

## Materials and methods

Photographs of the adults were taken by an Olympus E-M1 digital camera with a M. ZUIKO DIGITAL ED 60 mm F2.8 Macro lens. To examine the male genitalia, the abdomen was removed and soaked in 10% potassium hydroxide solution at room temperature for about 24 hours and was dissected under a Nikon SMZ18 stereoscope, following Hu et al. (2018). The genitalia preparation was photographed by a Nikon D7500 digital camera mounted to the stereoscope. All photographs were adjusted in Adobe Photoshop CC (Adobe Systems Inc., San Jose, CA, USA). Specimens are deposited in the insect collection, Department of Forest Protection, Beijing Forestry University (BFU), Beijing, China.

## Taxon treatments

### Drupadia scaeva

(Hewitson, 1869)

D9D8C9F4-C372-5584-9389-C7C2C4618AC3

#### Materials

- **Type status:** Other material. **Occurrence:** recordedBy: Si-Xun Ge; Zhuo-Heng Jiang; individualCount: 3; sex: 3 males; lifeStage: adult; **Taxon:** scientificName: *Drupadia scaeva* (Hewitson, 1869); kingdom: Animalia; phylum: Arthropoda; class: Insecta; order: Lepidoptera; family: Lycaenidae; genus: Drupadia; specificEpithet: *scaeva*; taxonRank: species; scientificNameAuthorship: (Hewitson, 1869); **Location:** country: China; stateProvince: Yunnan Province; county: Mengla Country; locality: Menglun township; maximumElevationInMeters: 800 m a.s.l.; verbatimCoordinates: 21°9629′ N, 101°2073′ E; **Identification:** identifiedBy: Shao-Ji Hu; dateIdentified: 2021; **Event:** samplingProtocol: sweep net; year: 2021; month: 4; day: 24; habitat: Rain Forest; **Record Level:** basisOfRecord: PreservedSpecimen

#### Description

**Figure 1.**
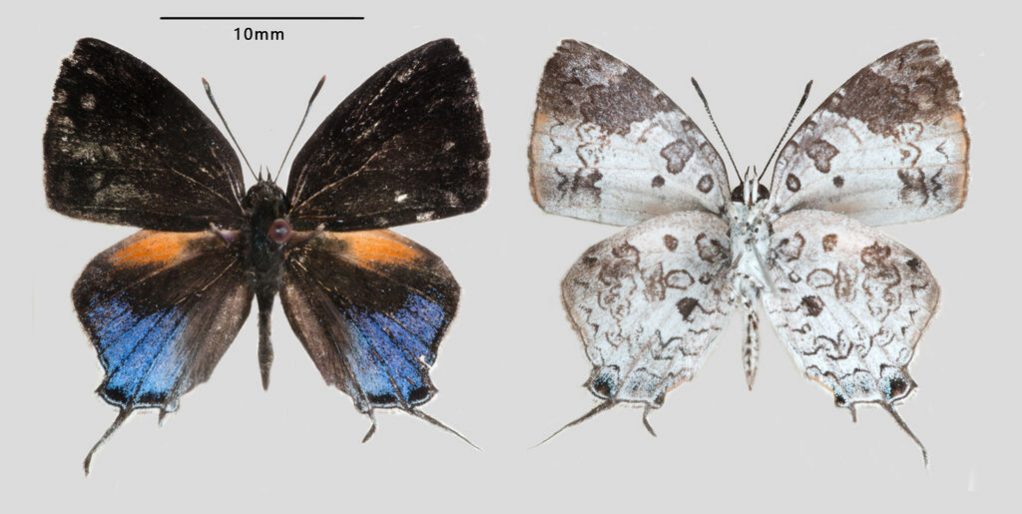
Male of *Drupadia scaeva* (Hewitson, 1869) collected in Menglun Township, Mengla County, Xishuangbanna, Yunnan, China.

**Male** (Fig. 1): Fore-wing length 12 mm. Upperside: Fore-wing entirely blackish-brown. Hind-wing blackish-brown with oval orange patch on the costal margin. The lower part of hind-wing iridescent blue, with two black spots surrounded by whitish lines distally in spaces 2 and 1b. Tails blackish with white tips. Underside: Both wings greyish-white with scattered irregular dark markings. Fore-wing with large greyish sub-quadrilateral apical spot; outer marginal area from veins M3 to 2A pale orange, with three dark parallel wavy striae narrowly developed. Two basal spots in the discocell and a small dark spot distinctly developed within the space 1b proximally. Hind-wing whitish, with delicate dark spots and stripes. The greyish apical spot large, with scattered wavy striae; five irregular spots present in tornal area; dark markings at the ends of both discocells strongly developed as parallel wavy striae; two tornal spots in spaces 2 and 1a, with light bluish scales in spaces 1a, 1b and 2.

**Figure 2.**
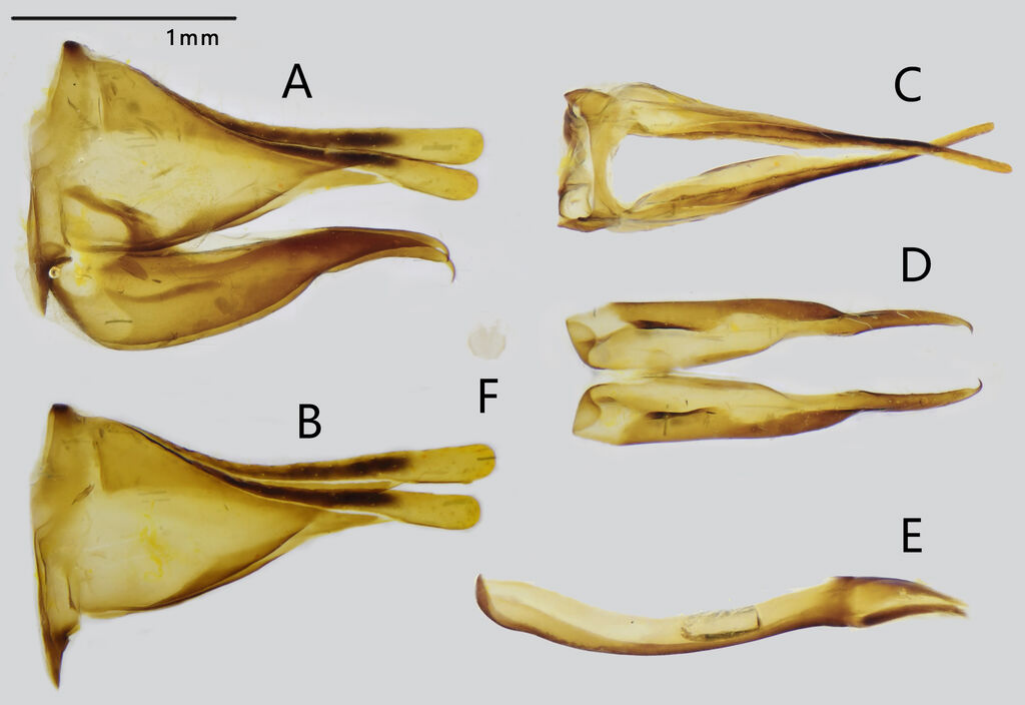
Male genitalia of *D. scaeva*: **A.** entire genitalia (except aedeagus); **B.** lateral view of ring and tegumen; **C.** dorsal view of tegumen; **D.** ventral view of valves; **E.** lateral view of aedeagus; **F.** posterior view of juxta.

**Male genitalia** (Fig. 2): Highly sclerotised. Ring straight of moderate width; tegumen extends into two triangular-shaped long lobes, elongated as flattened tips distally in the lateral view. Valve triangulate, rather elongated distally with a slightly curved pointed tip. Aedeagus rather strong, dorsally curved with a radian in the lateral view. Juxta small, round disc-shaped in posterior view.

#### Distribution

Myanmar; Thailand; Singapore; India; Bhutan; Laos; Vietnam; China (new record)

#### Notes

We tentatively identified specimens collected in this study as ssp. cooperi, with the bluish scales in the centre of fore-wings absent and a relatively limited violet area on hind-wings.

### Tajuria sekii sisyphus

Ge & Jiang n.

566CA7A0-CD39-5557-A79A-2406410BBD06

69624BFA-969D-44FC-9ED6-CFC6BEB9E2B5

#### Materials

- **Type status:** Holotype. **Occurrence:** recordedBy: Si-Xun Ge; individualCount: 1; sex: male; lifeStage: adult; disposition: in collection; **Location:** country: China; countryCode: CN; stateProvince: Yunnan; county: Mengla Country; locality: Menglun township; verbatimElevation: 800 m; verbatimCoordinates: 21°9629′ N, 101°2073′ E; **Identification:** identifiedBy: Si-Xun Ge; dateIdentified: 2021; **Event:** samplingProtocol: sweep net; year: 2021; month: 4; day: 24; habitat: Rain Forest; **Record Level:** basisOfRecord: PreservedSpecimen
- **Type status:** Paratype. **Occurrence:** recordedBy: Si-Xun Ge; Zhuo-Heng Jiang; individualCount: 4; sex: 4 females; lifeStage: adult; disposition: in collection; **Location:** country: China; countryCode: CN; stateProvince: Yunnan; county: Mengla Country; locality: Menglun township; verbatimElevation: 800 m; verbatimCoordinates: 21°9629′ N, 101°2073′ E; **Identification:** identifiedBy: Si-Xun Ge; dateIdentified: 2021; **Event:** samplingProtocol: sweep net; year: 2021; month: 4; day: 24; habitat: Rain Forest; **Record Level:** basisOfRecord: PreservedSpecimen

#### Description

**Figure 3.**
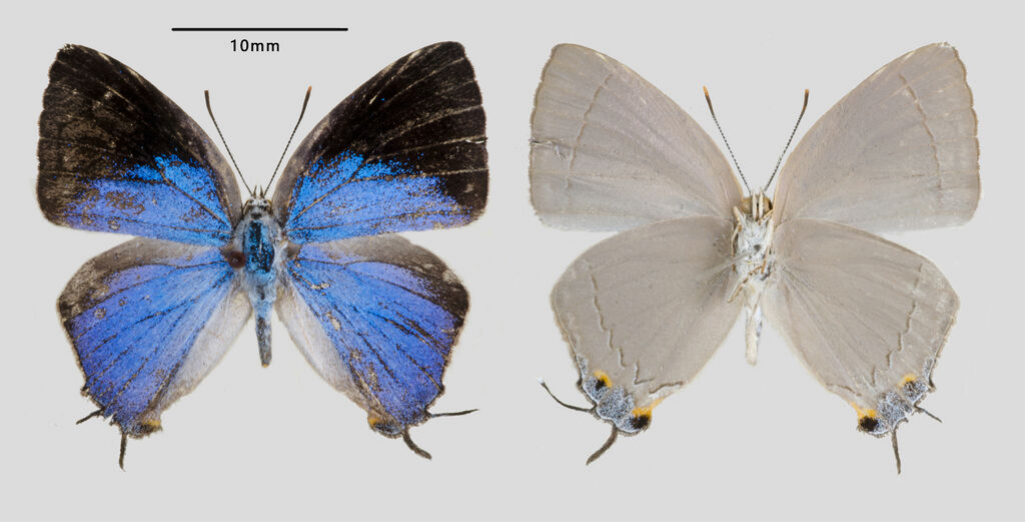
*Tajuria sekii sisyphus* ssp. nov., (Holotype, male).

Male (Fig. 3): Fore-wing length 13 mm. Upperside: Both wings shiny royal blue, fore-wing with veins black partly suffused with blue scales. Black border broadening at apex rather developed, from costal to tornal angle with most parts of space 2 blackish and space 3 completely blackish. Hind-wing ground colour as in fore-wing with costal margin pale brownish and extremely narrow blackish border; tails blackish with white tips; orange spot about 1/3 of tornal lobe or less, with caudal portion greyish. Underside: Pale greyish-brown, with narrow dark brownish post-discal striae almost parallel to outer margins on both wings. Dark markings at ends of both discocells absent. Tornus of hind-wing grey, with distinct black spots in spaces 2 and 1a crowned by prominent orange proximally.

**Figure 4.**
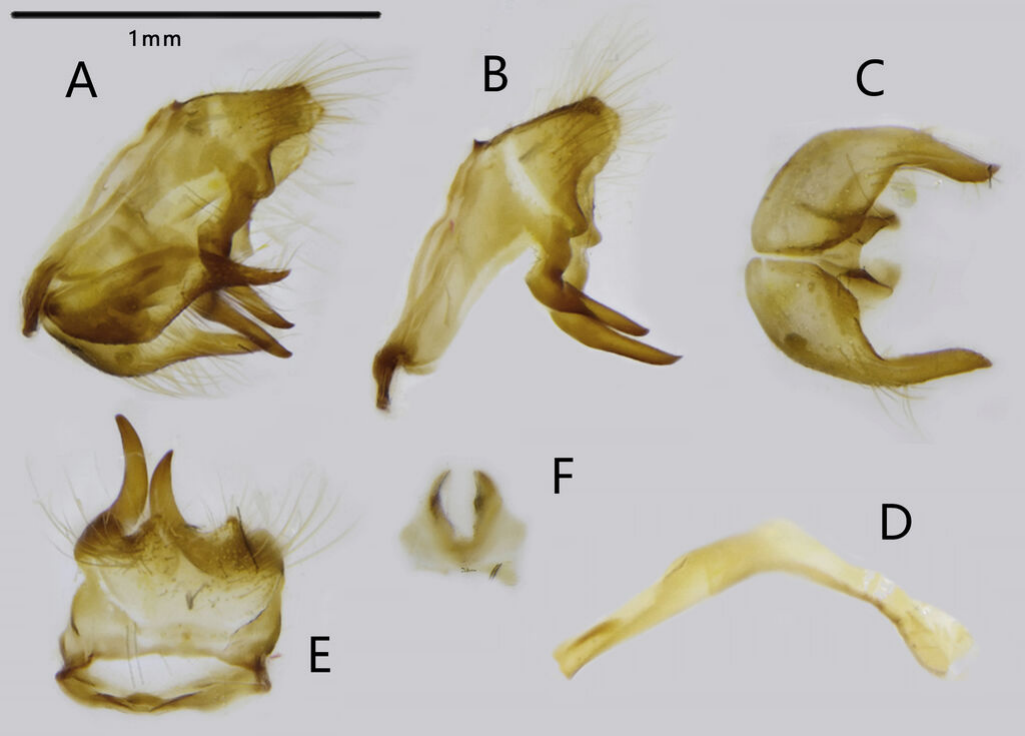
Male genitalia of *T. sekii sisyphus* ssp. nov.: **A.** entire genitalia (except aedeagus); **B.** lateral view of ring and tegumen; **C.** ventral view of valves; **D.** lateral view of aedeagus; **E.** dorsal view of tegumen; **F.** posterior view of juxta.

Male genitalia (Fig. 4): Highly sclerotised. Ring straight and broad in width, extends into two falx-shaped lobes distally; tegumen broad and short, with two lobes, a pair of blunt teeth with long setae in dorsal view and truncated end in lateral view. Valve quadrilateral, elongated distally with pointed tip covered by long sparse setae. Aedeagus slender and ventrally curved in the middle. Juxta hoof-shaped in posterior view.

**Figure 5.**
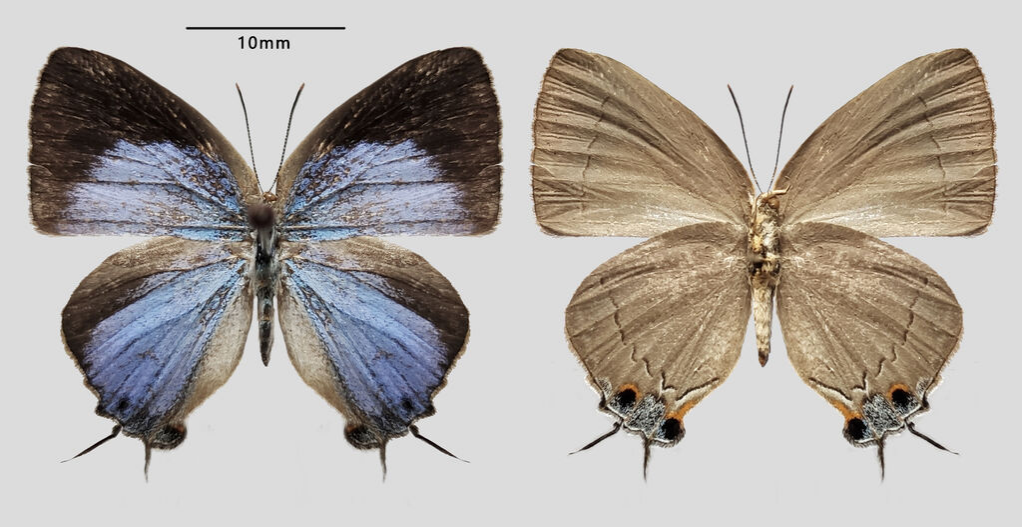
*Tajuria sekii sisyphus* ssp. nov., (Paratype, female).

Female (Fig. 5): Fore-wing length 13 mm. Resembles male, but with blue area relatively more reduced and wing shape broader and more rounded, hind-wing with tornal black spot in space 2.

#### Diagnosis

*Tajuria sekii sisyphus* ssp. nov. can be distinguished from the nominotypical subspecies by the combination of the following characteristics: 1) Blue areas of both sexes are more reduced, especially in female, 2) Hindwing with tornal black spot in space 2 on the upperside of females, while absent in nominotypical subspecies and 3) Compared to the nominotypical subspecies, females of the new subspecies with both wings broader and more rounded.

#### Etymology

The subspecies name is derived from the name of a man in Greek mythology, who also appears in *The Myth of Sisyphus*. The process of scientific research is just like the act of rolling the boulder up the hill. However, the description of each new taxon is exciting enough. "Each atom of that stone, each mineral flake of that night-filled mountain, in itself forms a world. The struggle itself toward the heights is enough to fill a man's heart. One must imagine Sisyphus happy." The subspecies name is treated as a noun in apposition.

#### Distribution

The nominotypical subspecies can be found in Thailand, Vietnam and Laos, while ssp. sisyphus is only known from China.

## Discussion

China is a country with mega-biodiversity of butterflies, especially the southwest mountainous part (Wang et al. 2020). Yunnan Province harbours great species richness with a lot of new taxa and new records of lycaenid butterflies discovered in recent times (Hu and Zhang 2009, Hu and Zhang 2010, Hu et al. 2012a, Hu et al. 2012b, Zhang et al. 2020).

Before this contribution, photos of *D. scaeva* had already been taken by nature lovers in Xishuangbanna Prefecture, while there has been no official publication of this species in China. In this expedition, specimens of *D. scaeva* were collected and it was confirmed that the species is, indeed, present in China.

The genus *Tajuria* has a high diversity in Oriental and Australian Realms. However, systematic studies on this genus are still very limited, even in recent years; therefore, the definition of some species is still unresolved. We speculate that there should be more species of *Tajuria* to be found in China.

## Supplementary Material

XML Treatment for Drupadia scaeva

XML Treatment for Tajuria sekii sisyphus
